# Altered expression of kisspeptin, dynorphin, and related neuropeptides in polycystic ovary syndrome: A cross-sectional study

**DOI:** 10.18502/ijrm.v22i5.16440

**Published:** 2024-07-08

**Authors:** Andon Hestiantoro, Rachellina Noor Al Maghfira, Ratna Fathmasari, Ririn Rahmala Febri, Ericko Ongko Joyo, Raden Muharam, Gita Pratama, Anom Bowolaksono

**Affiliations:** ^1^Reproductive Immunoendocrinology Division, Department of Obstetrics and Gynecology, Faculty of Medicine, University of Indonesia/Cipto Mangunkusumo General Hospital, Jakarta, Indonesia.; ^2^Cluster of Human Reproduction, Fertility and Family Planning, Indonesia Medical Education and Research Institute, University of Indonesia, Jakarta, Indonesia.; ^3^Department of Biology, Faculty of Mathematics and Natural Sciences, University of Indonesia, Depok, Indonesia.; ^4^Faculty of Medicine University of Indonesia, Jakarta, Indonesia.; ^5^Cellular and Molecular Mechanisms in Biological System Research Group, Department of Biology, Faculty of Mathematics and Natural Sciences, University of Indonesia, Depok, Indonesia.

**Keywords:** Dynorphins, Kisspeptins, Neuropeptides, Polycystic ovary syndrome.

## Abstract

**Background:**

Since kisspeptin (*KISS1*) in the hypothalamus is affected by the inhibitory effect of dynorphin, it raises questions about the controlled balance of these 2 neuropeptides in women with and without polycystic ovary syndrome (PCOS).

**Objective:**

This study compares the expression levels of *KISS1*, dynorphin, neurokinin-B, leptin, and neuropeptide-Y in women with and without PCOS.

**Materials and Methods:**

In this cross-sectional study, the peripheral blood samples of 20 women with PCOS and 20 women without PCOS who referred to Yamin Kencana Clinic, Cipto Mangunkusumo hospital, Jakarta, Indonesia were enrolled from August-December 2022. mRNA relative expression of genes related to the central factors associated with PCOS, such as leptin, neuropeptide-Y, *KISS1*, tachykinin-3, and prodynorphin (*PDYN*), in PCOS and non-PCOS populations were examined. Gene quantification was carried out by the quantitative polymerase chain reaction method.

**Results:**

The *KISS1*/*PDYN* ratio was significantly higher in the PCOS group than in the control group (p = 0.02), and the *PDYN* was lower in the PCOS group than the control group (p < 0.001). Moreover, the positive correlation between *KISS1* and the *KISS1*/*PDYN* ratio was significantly stronger in the PCOS group than in the control group (R = 0.93; p < 0.001 vs. R = 0.66, p < 0.001).

**Conclusion:**

Our results suggest that an increased *KISS1/PDYN* ratio in PCOS women is related to diminished dynorphin expression. Low expression of the gene encoding dynorphin and a high *KISS1*/*PDYN* ratio is highly specific to PCOS.

## 1. Introduction

The identification of the kisspeptin-neurokinin-B-dynorphin (KNDy) neuron network within the hypothalamus has provided valuable insights into the intricate roles played by these neurons in the pathogenesis of polycystic ovary syndrome (PCOS). PCOS is the most common endocrinopathy in women of reproductive age. It is associated with abnormal uterine bleeding and infertility due to ovulation disorders and abnormal androgen production. Diagnostic criteria for PCOS based on the 2003 Rotterdam consensus include hyperandrogenism, oligomenorrhea/anovulation, and polycystic ovarian morphology (1). Increased pulse amplitude and frequency of gonadotropin-releasing hormone (GnRH) in the hypothalamus cause relatively persistent and non-pulsatile luteinizing hormone (LH) secretion, resulting in chronic anovulation and hyperandrogenism, which leads to PCOS (2). The amplitude and frequency of GnRH pulsatile secretion are affected by several neuropeptides, such as kisspeptin (*KISS1*), neurokinin-B (NKB), and dynorphin, which are expressed by the *KISS1*, tachykinin-3 (*TAC3*), and prodynorphin (*PDYN*) genes, respectively (3). The discovery of the KNDy neuron network in the hypothalamus helps us understand the role of these neurons in regulating GnRH secretion at puberty and during reproductive age.

Numerous studies have investigated the genetic components of PCOS (4–9); for instance, Chaudhary et al. reveal that polymorphism in several genes play a role in the steroidogenesis pathway results in the phenotypic expression of PCOS (4). In another study, Farsimadan et al. found that polymorphisms within *KISS1* were associated with an increased risk of PCOS development (5). A recent meta-analysis also reported higher serum *KISS1* levels in subjects with PCOS than those without (6). Any disturbance in *KISS1* regulation also can lead to a metabolic imbalance, such as obesity (7). Moreover, hormonal profile differences in women with PCOS are still unclear (8, 9). Differences in hormone profiles in PCOS women may be related to differences in leptin (*LEP*) and neuropeptide-Y (*NPY*) gene expression levels. These recent studies enhance our understanding of the genetic basis of PCOS and shed light on the potential contributions of these genes to the development of the syndrome.

While some studies have measured the serum levels of neuropeptides in PCOS women (4–9), few studies have focused on the expression of the genes encoding these neuropeptides in peripheral blood (6, 10). Previous studies have stated that the mRNA expressions of these genes were detected in peripheral blood (11–13). Since the secretion of *KISS1* is influenced by many factors, such as dynorphin, *NKB*, *LEP*, and *NPY*, it would be interesting to study their role in the context of women with PCOS.

Knowledge of gene expression levels in women with PCOS is required to identify gene variation and its effect on phenotypic hormone variations as a basis for further research. This study aimed to compare *KISS1*, *PDYN*, *TAC3*, *LEP*, and *NPY* expression levels in women with and without PCOS.

## 2. Materials and Methods

### Subjects

In this cross-sectional study, we consecutively included 40 women of reproductive age (15–49 yr), consisting of 20 diagnosed with PCOS and 20 without PCOS, who were referred to Yamin Kencana Clinic, Cipto Mangunkusumo hospital, Jakarta, Indonesia from August to December 2022. The diagnosis of PCOS was determined using the Rotterdam 2003 criteria, which includes the presence of at least 2 of the following: a) oligo-ovulation or anovulation, b) clinical or biochemical signs of hyperandrogenism, and c) polycystic ovaries on ultrasound examination (14). Women diagnosed with PCOS were included in the PCOS group, while women without PCOS served as the control group (n = 20/each). Participants were excluded if they were either pregnant, on medications that could alter metabolic parameters for the past 2 months, or had endocrine abnormalities.

### Sample size

The sample size was calculated using the Lemeshow formula. The prevalence of PCOS in Indonesia is 8–10%, so the proportion value of 10% is used. The percentage or errors used in this study was 5% ( α = 5%). The precision used in this was 20% (d = 0.2). Based on these calculations, 10 samples are sufficient for each group. However, we used 20 participants in each group for the power of our study.

### Sample collection and processing

Blood samples were collected at the Yamin Kencana Clinic, Cipto Mangunkusumo hospital, Jakarta, Indonesia following the World Health Organization standard blood collection procedure (15). Sample processing was conducted at the Human Reproductive, Infertility, and Family Planning Laboratory of the Indonesian Medical Education and Research Institute, Faculty of Medicine, University of Indonesia, Jakarta. The authors confirm that all experiments were performed in accordance with relevant guidelines and regulations.

Gene quantification was carried out by the quantitative polymerase chain reaction method using the SensiFAST SYBR Green PCR Master Mix [Bioline] kit and Prime Pro 48 quantitative polymerase chain reaction system from Cole-Parmer Ltd [TechneTM] polymerase chain reaction machine based on the given protocols. The housekeeping gene used in this study was β -actin. The primary base sequence of the *LEP*, *NPY*, *KISS1*, *TAC3*, *PDYN*, and β -actin genes was obtained from the National Center for Biotechnology Information database. Relative gene expressions of each gene were obtained by processing the cycle threshold (Ct) value with the Livak method (16).

The Livak method calculates the relative gene expression by comparing the expression of target genes with reference genes. The reference gene Ct normalized target gene Ct and control gene Ct, resulting in Δ Ct ${\;}_{\text{target}}$ and Δ Ct ${\;}_{\text{control}}$ , respectively. Then Δ Ct ${\;}_{\text{target}}$ was subtracted against the Δ Ct ${\;}_{\text{control}}$ to obtain the ΔΔ Ct. The value obtained was a relative comparison to the control using a 2-based exponential (2 ${\;}^{-\Delta \Delta \text{Ct}}$ ).

In addition to assessing relative gene expression, this study measured hormone levels as demographic variables. Specifically, testosterone, sex hormone-binding globulin (SHBG), and free androgen index were measured to evaluate hormonal profiles in women with and without PCOS. Peripheral venous samples were collected and subjected to centrifugation to obtain serum for the quantitative measurement of testosterone and SHBG concentrations. The enzyme-linked immunosorbent assay method was employed for this purpose. Commercial fluorescence enzyme immunoassay kits were used for the determination of serum testosterone (ST AIA-Pack Testosterone, TOSOH, Japan, Cat. No. 0025204) and SHBG (ST AIA-Pack SHBG, TOSOH Bioscience, Japan), following the manufacturer's instructions. The assay utilized a sandwich-type approach with a pre-coated 96-well plate and enzyme-labeled secondary antibodies. A sample volume of 300 µl was required for analysis. Before measurement, each sample was labeled and prepared in a sample cup and a test cup. The prepared sample and test cups were then inserted into the TOSOH instrument. The free androgen index was calculated as the ratio of total testosterone to SHBG and reported as a percentage. Baseline demographic characteristics and hormonal profiles were compared to see if there were any significant differences between groups that could interfere with the interpretation of the study results. Potential confounding variables, such as age, body mass index, and hormonal level, were identified. Potential confounding variables due to disease and metabolic abnormalities were controlled using the exclusion criteria.

### Ethical considerations

All research participants gave their permission to be part of this study in the form of written informed consent. The Ethics Committee of the Indonesia and Cipto Mangunkusumo hospital, Jakarta, Indonesia has approved the study (Code: 0449/UN2.F1/ETIK/2018).

### Statistical analysis

Data analysis was performed using SPSS, version 24.0 (IBM Corp., Armonk, New York, USA). Continuous data were presented as mean ± standard deviation (SD) or median and interquartile ranges (IQR) as appropriate. The Student's *t* test was used to compare the 2 groups' means if the data distribution was normal. In cases in which the data distribution was not normal, the Mann-Whitney test was used for the comparative analysis. Pearson's correlation analysis was performed to analyze the correlation of the relative expression of *KISS1* to the *KISS1*/*PDYN* and *KISS1*/*TAC3* ratio with a significant level of < 0.05.

## 3. Results

### Demographic and hormonal profiles

For this study, a total of 40 women were consecutively screened for eligibility based on the inclusion and exclusion criteria, and none of them were found to be ineligible or refused to participate. Finally, the analysis included 20 women in the PCOS and 20 women in the control group.

The mean age of the participants was 30.00 ± 5.00 yr in the PCOS and 28.83 ± 1.25 yr in the control group. The 2 groups had no significant differences in age, body mass index, and hormonal profiles, except for a longer menstrual cycle in the PCOS group. Moreover, no significant difference was observed in the total testosterone levels, SHBG levels, and free androgen index in the 2 groups (Table I).

### Comparison of *KISS1, PDYN, TAC3, LEP,* and *NPY* mRNA levels

No significant differences were revealed in the relative expression of the *KISS1*, *TAC3*, *LEP*, *NPY*, kisspeptin-to-tachykinin-3 ratio (*KISS/TAC3*), and *LEP*-to-*NPY* ratio (*LEP*/*NPY*) between the PCOS and control groups (Table II). However, *PDYN* relative expression was significantly lower in the PCOS group than in the control group (0.79 [1.79] vs. 1.65 [2.82]; p < 0.001). This was followed by a significant difference in the kisspeptin-to-dynorphin (*KISS1*/*PDYN*) ratio (2.35 [4.35] vs. 1.30 [1.15]; p = 0.02).

### Correlation between *KISS1* and its ratio to dynorphin and NKB

We sought the correlation of the relative expression of *KISS1* to the *KISS1*/*PDYN* and *KISS1*/*TAC3* ratio to reveal the controlled balance of these neuropeptides since *KISS1* in the hypothalamus is affected by the inhibitory effect of dynorphin and the stimulatory effect of NKB. By comparing the relative expression of *KISS1* against dynorphin and NKB, we get a ratio that reflects the controlled balance of these 2 neuropeptides in the hypothalamus.

**Figure 1:**
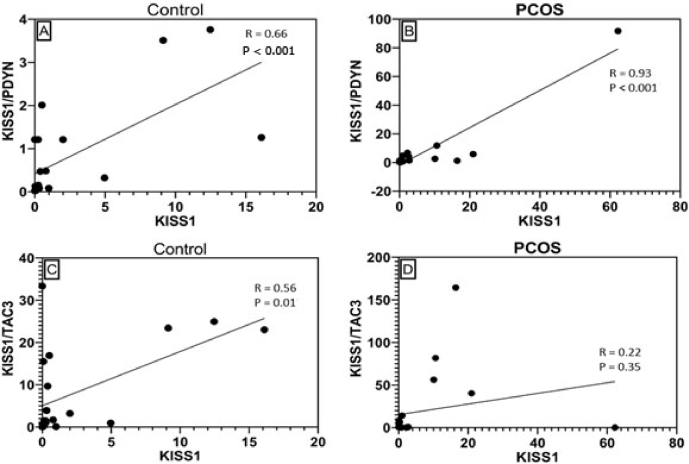
Correlation between kisspeptin-to-prodynorphin ratio in the A) Control and B) PCOS groups, kisspeptin-to-tachykinin-3 ratio in the C) Control and D) PCOS groups. Pearson correlation test.

Correlation analysis was performed separately for the control and PCOS groups (Figure 1). Pearson's correlation test found a significant positive correlation between *KISS1* mRNA expression and the *KISS1*/*PDYN* ratio. The correlation of *KISS1* and *KISS1*/*PDYN* ratio was revealed to be stronger in the PCOS group (Pearson R = 0.93, p < 0.001) than in the control group (Pearson R = 0.66, p < 0.001). On the other hand, a positive and significant correlation was observed between *KISS1* and the *KISS1*/*TAC3* ratio in the control group (Pearson R = 0.56, p = 0.01) but was not found in the PCOS group (Pearson R = 0.22, p = 0.35).

**Table 1:** Demographic and hormonal profiles of subjects in the PCOS and control groups


**Variables** * *	**Control (n = 20)**	**PCOS (n = 20)** * *	**P-value**
**Age (yr)** * *	30.00 ± 5.00 (30.00, 8.00)	28.83 ± 1.25 (29.00, 5.00)	0.61*
**Body mass index (kg/m^2^)** * *	24.19 ± 4.16 (24.40, 7.43)	26.26 ± 5.48 (26.15, 8.00)	0.19*
**Weight (kg)** * *	59.37 ± 10.41 (59.00, 14.00)	61.00 ± 28.50 (61.00, 26.00)	0.20*
**Height (m)** * *	1.57 ± 0.04 (1.56, 0.10)	1.57 ± 0.63 (1.57, 0.61)	0.84*
**Menstrual cycle (days)** * *	30.00 ± 5.00 (30.00, 5.00)	50.00 ± 71.00 (50.00, 56.00)	< 0.001**
**Testosterone (nmol/l)** * *	1.22 ± 4.05 (1.16, 3.65)	1.66 ± 1.22 (1.55, 1.55)	0.85**
**SHBG (nmol/l)** * *	77.70 ± 67.76 (69.80, 100.29)	49.48 ± 75.46 (43.96, 60.23)	0.16**
**Free androgen index (%)** * *	1.69 ± 7.21 (1.55, 6.29)	2.64 ± 6.82 (3.15, 5.99)	0.24**
Data presented as Means ± SD (median, IQR). *Student's *t* test. **Mann-Whitney test.PCOS: Polycystic ovary syndrome,SHBG: Sex hormone-binding globulin			

**Table 2:** Comparison of mRNA relative expression between PCOS and control groups


**Variables**	**Control (n = 20)**	**PCOS (n = 20)**	**P-value**
***LEP***	1.81 ± 1.18 (1.48, 1.95)	2.95 ± 2.21 (2.45, 2.68)	0.09*
***NPY***	1.28 ± 1.85 (1.28, 1.78)	1.14 ± 1.02 (1.32, 1.06)	0.74**
***KISS1***	0.28 ± 1.67 (0.28, 3.88)	0.62 ± 8.16 (0.62, 8.16)	0.21**
***TAC3***	1.00 ± 3.16 (0.20, 3.17)	1.48 ± 4.44 (0.16, 4.53)	0.19**
***PDYN***	2.55 ± 2.99 (1.65, 2.82)	1.03 ± 1.83 (0.79, 1.79)	< 0.001**
***LEP*** **/** ***NPY*** ** ratio**	1.09 ± 3.55 (0.94, 3.19)	2.62 ± 3.38 (2.35, 3.13)	0.19**
***KISS1*** **/** ***PDYN*** ** ratio**	0.32 ± 1.13 (1.30, 1.15)	1.00 ± 2.84 (2.35, 4.35)	0.02**
***KISS1*** **/** ***TAC3*** ** ratio**	1.58 ± 15.8 (1.58, 15.88)	0.86 ± 12.33 (0.86, 12.33)	0.99**
Data presented as Means ± SD (median, IQR). *Student's *t *test. **Mann-Whitney test. *KISS1*: Kisspeptin, *LEP*: Leptin, *NPY*: Neuropeptide-Y, *PDYN*: Prodynorphin, *TAC3*: Tachykinin-3,PCOS: Polycystic ovary syndrome			

## 4. Discussion

In this study, we could not establish a higher *KISS1* expression in the PCOS group than in the control group. The level of protein or peptide contained in the body significantly and positively correlated with the mRNA expression of its genes (17). Despite the absence of studies that have determined the *KISS1* mRNA expression in PCOS women, the results of our study are in line with the studies on *KISS1* serum levels. Several recent publications reported higher serum levels of *KISS1* in subjects with PCOS than in controls (6, 18–22), while 2 reported the contrary (10, 23). Ibrahim et al. report that serum levels of *KISS1* were significantly higher in PCOS women than in the control group (22).

Previous research suggests that *KISS1* is secreted in a pulsatile manner from the hypothalamus and peripheral organs such as the pancreas, ovaries, adipose tissue, and adrenal glands (24). In contrast to *KISS1*'s intrahypothalamic functions, little is known about *KISS1*'s physiological importance in extrahypothalamic tissues. *KISS1* appears to function in the peripheral systems that control reproduction in mammals, but more research is needed (25). The current knowledge does not rule out the potential that extrahypothalamic *KISS1* acts as an autocrine/paracrine factor in the peripheral tissues to fulfill its physiological function. Furthermore, *KISS1* neurons' development and function can be influenced by metabolic factors at various stages of development. The metabolic changes that *KISS1* neurons undergo can be temporary or permanent (for example, epigenetic modifications) (26). This makes it evident that peripheral changes can affect the increase in hypothalamic *KISS1* production in PCOS.

This study's foremost, and novel finding was that dynorphin expression was lower in typical PCOS women than in controls. These results demonstrated a significantly higher *KISS1*/*PDYN* ratio in PCOS women than in controls. To our knowledge, a similar finding regarding lower dynorphin levels in PCOS women has not been published before. Research on these genes in the pathophysiology of PCOS has not been widely carried out. Although no studies have examined dynorphin levels or the *KISS1*/*PDYN* ratio in PCOS women, our findings align with the basic theory of regulatory systems in which KNDy neurons regulate GnRH pulsatility. It is known that *KISS1*, while the inhibitory function of dynorphin on *KISS1,* works to control *KISS1* secretion with NKB as a balance regulator. Thus, reduced dynorphin production will undoubtedly have an impact on increasing *KISS1* secretion, which in turn causes an increase in the frequency and amplitude of the pulsatility of GnRH secretion (27).

Furthermore, we also found a significant correlation between the *KISS1* and *KISS1*/*PDYN* ratio, which was stronger in the PCOS groups than in the control groups. This is thought to be related to the reduction of dynorphin expression, which then affects the increase in GnRH pulsatility as the pathophysiology of PCOS. Meanwhile, the correlation between *KISS1* and *KISS1/TAC3* was weaker and non-significant in the PCOS groups. These different correlation findings indicate that increased *KISS1* in PCOS women is not caused by increased NKB expression. However, it is more strongly associated with diminished dynorphin expression. Therefore, the diminished dynorphin expression in PCOS women seems to have a significant effect on *KISS1*, proven by the finding of a significant correlation, thus masking the effect of NKB, indicated by the correlation of *KISS1* and *KISS1/TAC3* ratio, which was not significant in the PCOS groups but in the control groups.

Another critical pathological feature of PCOS is the disruption of progesterone-negative feedback to the GnRH neuronal network that inhibits LH and GnRH pulsation frequency (28). Progesterone receptors are not generally found on GnRH neurons, so it is suspected that another system transmits this action. Arcuate neurons express dynorphin, which exerts an inhibitory effect on GnRH and LH expression and is implied to mediate progesterone-negative feedback (29). The mechanism of disruption of progesterone-negative feedback occurs due to exposure to prenatal androgens in sheep, where there is a reduction in dynorphin expression without a change in *KISS1* expression, which causes a decrease in inhibition of KNDy neurons to progesterone, even though progesterone sensitivity is not impaired (30). Nevertheless, these findings do not necessarily apply to humans because the study was done on sheep. Our findings support the hypothesis that decreased dynorphin expression may disrupt negative progesterone feedback. This is indicated by the significant finding of lower *PDYN* expression in PCOS women in this study.

The main limitation of this study is that there is no evidence to clarify that alterations in mRNA levels of KNDy peptides in the blood accurately reflect the levels of gene expression in the hypothalamus. To obtain a more meaningful understanding of PCOS, the findings of this study must be supported by future studies that directly tie blood levels of KNDy peptides to those in the hypothalamus. Moreover, this study only observed a small number of subjects. Increasing the sample size in future studies would allow better intergroup analyses. Furthermore, peripheral blood testing is expected to be a potential non-invasive alternative diagnosis for PCOS.

## 5. Conclusion

The low expression level of the mRNA gene encoding dynorphin is highly specific for PCOS. These findings were followed by a high *KISS1*/*PDYN* ratio specific to the PCOS group. Furthermore, a significant positive correlation was established between *KISS1* and the *KISS1/PDYN* ratio. This study indicates that the expression of these neuropeptide genes in peripheral blood is associated with PCOS.

##  Data availability

Data supporting the findings of this study is available at RIN Dataverse at https://data.brin.go.id, reference number hdl: 20.500.12690/RIN/BK50G5.

##  Author contributions

Andon Hestiantoro contributed substantially to the conception and design of the study, subject recruitment, interpretation of data, drafting the manuscript, and final approval of the version to be published. Rachellina Noor Al Maghfira and Ratna Fathmasari contributed substantially to laboratory workup, acquisition of data, and data completion. Ririn Rahmala Febri contributed substantially to laboratory preparation, laboratory workup, sampling management, laboratory management, and data analysis. Ericko Ongko Joyo contributed substantially to data analysis, drafting the article, and finalization of the article. Raden Muharam and Gita Pratama contributed substantially in revising it critically for important intellectual content. Anom Bowolaksono contributed substantially in supervising laboratory workup, interpretation of data, design of laboratory workup, and revising the article. Each author agrees to be personally accountable for the entire work.

##  Conflict of Interest

The authors declare that there is no conflict of interest.
